# Bidirectional Activity of the *NWC* Promoter Is Responsible for *RAG-2* Transcription in Non-Lymphoid Cells

**DOI:** 10.1371/journal.pone.0044807

**Published:** 2012-09-11

**Authors:** Agnieszka Laszkiewicz, Lukasz Sniezewski, Monika Kasztura, Lukasz Bzdzion, Malgorzata Cebrat, Pawel Kisielow

**Affiliations:** Laboratory of Molecular and Cellular Immunology, Department of Tumor Immunology, Institute of Immunology and Experimental Therapy, Polish Academy of Sciences, Wrocław, Poland; National Institute on Aging, United States of America

## Abstract

The recombination-activating genes *(RAG-1* and *RAG-2)* encode a V(D)J recombinase responsible for rearrangements of antigen-receptor genes during T and B cell development, and *RAG* expression is known to correlate strictly with the process of rearrangement. In contrast to *RAG-1*, the expression of *RAG-2* was not previously detected during any other stage of lymphopoiesis or in any other normal tissue. Here we report that the CpG island-associated promoter of the *NWC* gene (the third evolutionarily conserved gene in the *RAG* locus), which is located in the second intron of *RAG-2*, has bidirectional activity and is responsible for the detectable transcription of *RAG-2* in some non-lymphoid tissues. We also identify evolutionarily conserved promoter fragments responsible for this bidirectional activity, and show that it is activated by transcription factor ZFP143. The possible implications of our findings are briefly discussed.

## Introduction

Recombination-activating gene 1 (*RAG-1*) and *RAG-2* are closely linked genes encoding two subunits of the lymphocyte-specific RAG recombinase, which is indispensable for the V(D)J recombination responsible for the diversity of immunoglobulins (Igs) and T cell receptors (TCRs) [1]–[5]. *RAG* expression is strictly controlled and occurs during well-defined developmental stages of T and B cells, when rearrangements of the TCRγ, TCRδ, TCRβ, TCRα, IgH and IgL chains occur [6]–[8]. Coordinated expression of *RAG-1* and *RAG-2* is due to the activity of *cis*-regulatory elements located in the region 90 kbp upstream of *RAG-2* [9]–[12]. In addition to developing lymphocytes, *RAG* expression has been observed in some tumors [13], and *RAG-1* expression was observed in the central nervous system [14]. In the genome, the *RAGs* are localized in their immediate vicinity and their open reading frames span single coding exons.

*NWC* is the third evolutionarily conserved gene located in the *RAG* locus [15]. The non-coding first exon of the *NWC* gene is located in the intron of *RAG-2*; the *NWC* gene promoter is located near the coding exon of *RAG-2*; and the two genes are divergently transcribed. The localization of the first exon (and therefore also the promoter) of the *NWC* gene is evolutionarily conserved among vertebrates. The *NWC* promoter is linked to an evolutionarily conserved CpG island and is active in non**-**lymphoid cells, where it drives constitutive expression of *NWC* [16]. In developing lymphocytes, the CpG island associated with the *NWC* promoter becomes methylated, the promoter is inactivated, and its function is taken over by the *RAG-1* promoter, which results in expression of hybrid *RAG-1/NWC* transcripts containing the first exon of the *RAG-1* gene [15]. In addition to the primary *NWC* promoter, we recently described a secondary promoter located downstream of *NWC* exon I, which drives ∼10 times lower expression of *NWC* compared to the main promoter [17]. The structure (GC-rich, CpG island-containing, TATA-less) and localization of the primary *NWC* promoter are typical for bidirectional promoters [18], suggesting that it may be responsible for the transcription of both *NWC* and *RAG-2*. We herein tested this possibility, and report for the first time that the *NWC* promoter has bidirectional activity, driving detectable expression of *RAG-2* in non-lymphoid tissues. We also identify two promoter elements capable of binding transcription factor ZFP143 and show that it activates the promoter.

## Results

### Identification of Bidirectional Activity of the *NWC* Promoter

**Figure 1:**
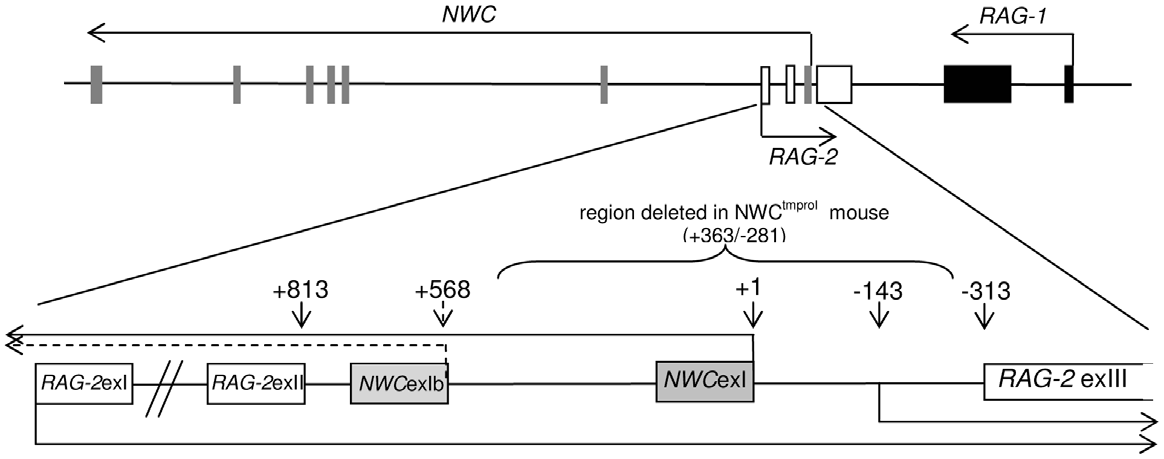
Organization of the mouse *RAG/NWC* locus and detailed structure of the region containing the *NWC* promoter. The relative positions of the exons encoding *RAG-1* (black boxes), *RAG-2* (open boxes), and *NWC* (gray boxes) are shown. Horizontal arrows indicate transcription start sites and orientations.

**Figure 2:**
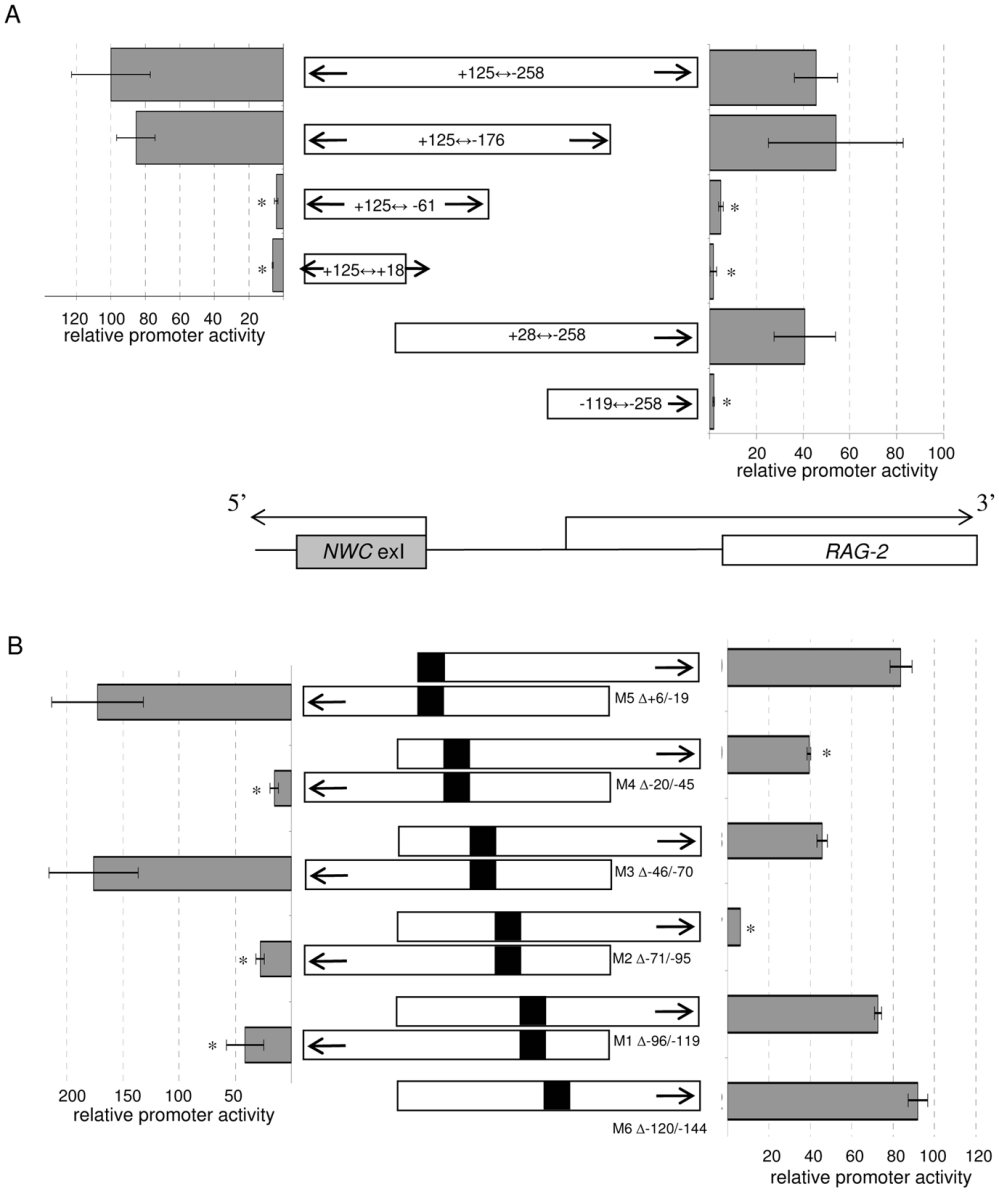
Characterization of the bidirectional activity of the *NWC* promoter by Dual-Luciferase Reporter (DLR) assays. A) Genomic fragments (represented schematically with open boxes at the center of the graph) were cloned into a firefly luciferase reporter vector in either the sense (*NWC*) or antisense (*RAG-2*) direction, as indicated with horizontal solids arrows. Numbers indicate positions relative to the *NWC* transcription start site. The activities of the promoter constructs were tested in NIH3T3 cells. The relative promoter activities are presented as a percent of the activity of the *NWC*-oriented +125/−258 fragment, which was taken as 100%. The results shown are the means of three to five experiments, with error bars representing ± 1 SD. Asterisks indicate significant differences (p < 0.05) in promoter activity between full-length (+125/−258) and truncated fragments. B) Activities of internal deletion mutants (M1–M6) of the −119/+125 and +28/−258 fragments cloned into a firefly luciferase reporter vector in the *NWC* and *RAG-2* orientations, respectively. The deletion ranges and their schematic representations (black boxes) are presented in the center of the graph. Numbers indicate positions relative to the *NWC* transcription start site. The relative promoter activities are presented as a percent of the activity of the +125/−119 (for *NWC*-oriented constructs) or +28/−258 (for *RAG-2*-oriented constructs) fragments, which were taken as 100%. The results shown are the means of three experiments, with error bars representing ± 1 SD. Asterisks indicate significant differences (p < 0.05) in promoter activity between the full-length constructs (+125/−119 or +28/−258) and the corresponding deletion mutants.

In the murine genome, the transcription start site (TSS) of the *NWC* gene and the beginning of the third exon of *RAG-2* are separated by 313 nucleotides (Fig. 1). The shortest fragment retaining promoter activity covers the region 119 nucleotides upstream of the *NWC* TSS [16]. To determine whether the *NWC* promoter has bidirectional activity, we cloned a genomic fragment spanning nucleotides +125/−258 relative to the *NWC* TSS in the forward (*NWC*) and reverse (*RAG-2*) directions upstream of the firefly luciferase gene contained in the pGL3-Basic reporter vector. As shown at the uppermost part of Figure 2A, Dual-Luciferase Reporter (DLR) analysis performed using NIH3T3 cells transfected with these vectors revealed that the +125/−258 fragment activated transcription in both orientations, demonstrating the bidirectional activity of this promoter. Thereafter, 5′-RACE performed on cells transfected with a reporter vector containing the +125/−258 fragment in the reverse direction allowed us to determine that *RAG-2*-oriented transcription starts at position −143 relative to the *NWC* TSS.

### Identification of Promoter Elements Responsible for the Bidirectional Activity of the *NWC* Promoter

To identify which fragments of the *NWC* promoter are responsible for its bidirectional activity, we inserted various portions of the genomic DNA localized between the third exon of *RAG-2* and the first exon of *NWC* into the pGL3-Basic reporter vector. The promoter activity of the inserted fragments was tested in the sense and antisense orientations, using DLR assay. As shown in Figure 2A, fragments +125/−258 and +125/−176 relative to the *NWC* TSS, which were active in the direction of *NWC* [16], were also active in the direction of the *RAG-2* gene. Consistent with this, fragments that failed to exhibit promoter activity in the direction of *NWC* (−125/+61 and −125/+18) also lacked activity in the direction of *RAG-2*. To narrow down the promoter region responsible for activity in the *RAG-2* direction, we tested fragments lacking the 5′ portions of the previously analyzed regions. We found that fragment +28/−258 retained promoter activity in the *RAG-2* direction, whereas fragment −119/−258 was inactive. Together, these results show that the previously described minimal fragment of the *NWC* promoter is sufficient for activity in the directions of both *NWC* and *RAG-2*. More detailed information on the *NWC* promoter elements required for its bidirectional activity was obtained by internal deletion of the −119/+125 and +28/−258 fragments, followed by activity analysis in the *NWC* and *RAG-2* directions, respectively. As shown in Figure 2B, the −71/−95 deletion down-regulated the promoter activity in both directions, whereas the −20/−45 deletion significantly decreased activity in the *NWC* direction but only slightly affected that in the *RAG-2* direction.

### The ZFP143 Transcription Factor Activates the *NWC* Promoter

**Figure 3:**
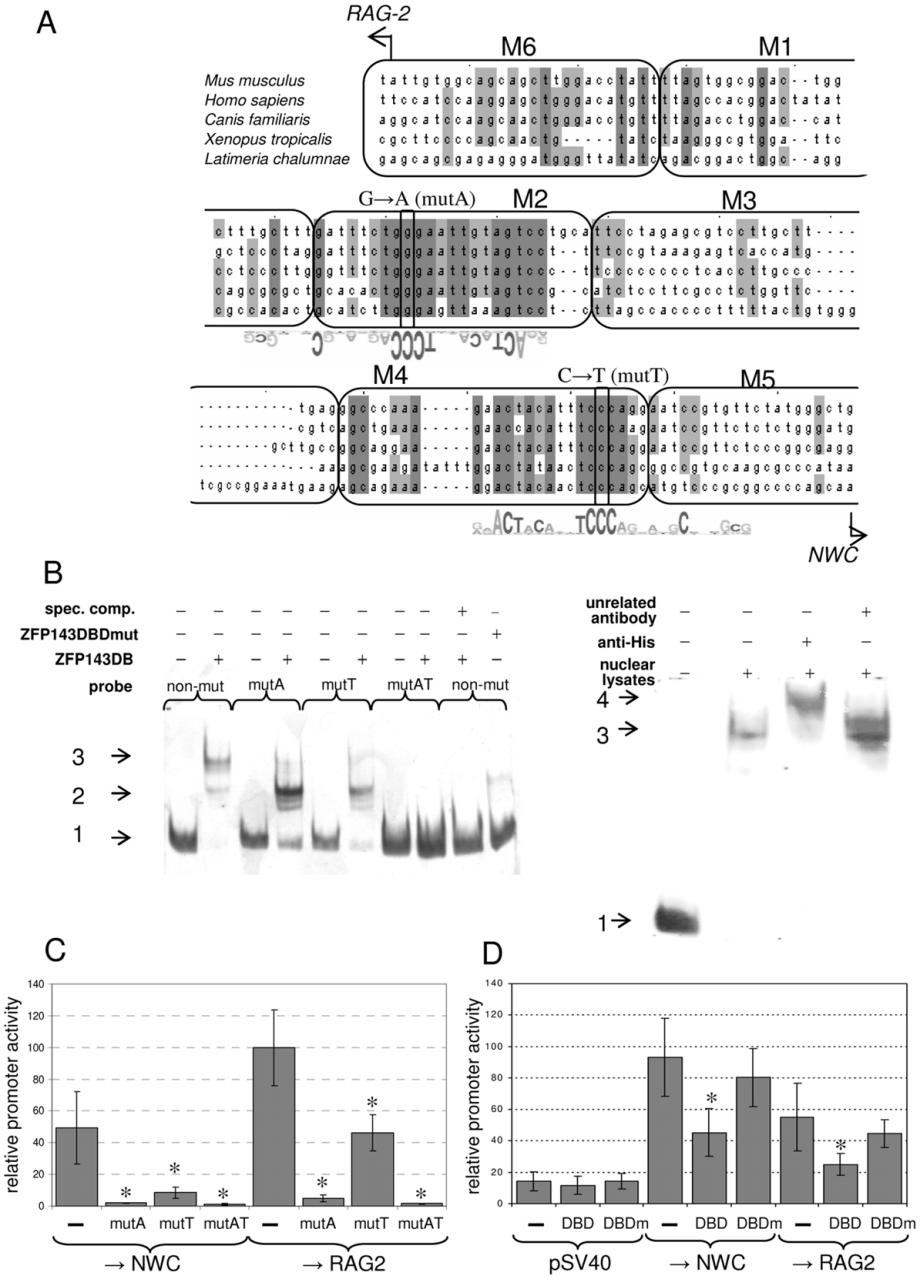
The ZFP143 transcription factor activates the *NWC* promoter. Comparison of vertebrate *NWC* promoter sequences. The ranges of the analyzed internal deletion mutants M1–M6 (rounded rectangles), transcription start sites of *RAG-2* and *NWC* murine transcripts (horizontal lines), and regions of 80% and 100% identity among analyzed species (light and dark gray shadings, respectively) are shown. The promoter sequence is aligned with ZFP143 sequence motifs representing the ZFP143 consensus binding site [19]. The positions and nature of the point mutations introduced into the putative ZFP143 binding sites of the *NWC* promoter are shown. EMSA experiments demonstrating binding of the ZFP143 protein to the *NWC* promoter. The left panel shows binding of purified recombinant ZFP143-DBD (wild-type and mutant) to non-mutated and mutated probes. The mutant of the ZFP143 DNA-binding domain was obtained by substituting the cysteines of the third and seventh zinc finger domains [20] with alanines. The right panel shows binding of the non-mutated probe to nuclear lysates obtained from cells expressing His-tagged full length ZFP143. Horizontal arrows indicate unbound probe (1), shifts consistent with binding to one (2) or both (3) ZFP143 binding sites, and the supershift obtained using an anti-His antibody (4). Abbreviations: spec**.** comp. – specific competitor; mutA, mutT and mutAT – mutant probes (see panel A), anti-His – anti-His antibody. C) Activity of *NWC* promoter fragments containing point mutations that disrupt the ZFP143 binding sites. Genomic fragments spanning nucleotides −119/+125 (for *NWC*-oriented constructs) or +28/−258 (for *RAG-2*-oriented constructs) relative to the *NWC* transcription start site containing one (mutA or mutT) or both (mutAT) mutations were cloned into a firefly luciferase reporter vector and tested in NIH3T3 cells. The relative promoter activities are presented as a percent of the activity of the intact (−) +28/−258 fragment, which was taken as 100%. Asterisks indicate significant differences (p < 0.05) in promoter activity between non-mutated and mutated fragments. D) Activity of *NWC* promoter fragments in the presence of a dominant-negative form of ZFP143. Firefly luciferase reporter vectors containing genomic fragments spanning nucleotides −119/+125 (*NWC*-oriented) or +28/−258 (*RAG-2*-oriented) relative to the *NWC* transcription start were co-transfected with ZFP143 DNA binding domain (DBD), mutant ZFP143 DNA binding domain (DBDm) or empty (–) expression vector. A reporter vector containing the SV40 promoter (pGL3-Prom) and a promoter-less vector (pGL3-Basic) were used as controls. The relative promoter activities are presented as a percent of the activity of pGL3-Basic co-transfected with the appropriate expression vector, which was taken as 1%. The results shown are means of three to four experiments with error bars representing ± 1 SD. Asterisks indicate significant differences (p < 0.05) in promoter activity in cells transfected with empty vector and the ZFP143-DBD expression vector.

The −71/−95 and −20/−45 fragments identified as being responsible for the activity of the *NWC* promoter correspond to highly conserved regions of the mammalian *NWC* promoters. We also noticed that these regions are imperfect inverted repeats separated by ∼40 bp and contain consensus binding sites for the transcription factor ZFP143 (RR**ACTACA**NN**TCCC**RNNNNN**C**NNGCG) (Fig. 3A), which is known to activate numerous bidirectional promoters [19]. Notably, these two consensus sequences are also present in non-mammalian species with sufficient reliable sequence data allowing us to localize the putative *NWC* promoter regions (*Xenopus tropicalis* and *Latimeria chalumnae*). As in the case of the mammalian promoters, the putative binding sites are localized 40–50 bp apart and form inverted repeats (Fig. 3A).

To test whether ZFP143 binds to its putative sites in the *NWC* promoter, we performed electromobility shift assays (EMSAs) using non-mutated promoter fragments (−119/+12 relative to the *NWC* TSS) and fragments containing point mutations disrupting one or both of the ZFP143 binding sites. Mutations were obtained by substituting thymine at the second cytosine of the most strictly required ZFP143 consensus sequence (TCCC). As shown in Figure 3B, the recombinant DNA-binding domain of ZFP143 (ZFP143-DBD) bound to the *NWC* promoter and formed one predominant shift, whereas binding to the single-mutant probes yielded a faster migrating complexes. This is consistent with binding to both (for the non-mutated promoter) or only one (for the single-mutant fragments) of the ZFP143 sites in the *NWC* promoter. Very faint shifted bands were observed when we used a double-mutant probe or a mutant form of the ZFP143-DBD peptide. We also performed EMSA experiments using nuclear extracts of cells expressing His-tagged full-length ZFP143 cDNA, and confirmed the specificity of the observed shift using an anti-6X His-tag antibody (Fig. 3B, right panel).

To test whether ZFP143 activates the *NWC* promoter, we first used DLR assay to analyze the activity of promoter fragments containing point mutations that disrupted the ZFP143 binding sites. As shown in Figure 3C, mutation of the site proximal to the *RAG-2* side of the promoter significantly decreased the promoter activity in both directions. In contrast, mutation of the site proximal to the *NWC* side of the promoter significantly decreased the promoter activity in the *NWC* direction but decreased that in the *RAG-2* direction by only ∼2-fold. These results are in very good agreement with those obtained using internal deletions of the promoter. Mutation of both sites further decreased the promoter activity in both directions to a level similar to that of the promoter-less vector. Because part of the putative ZFP143-binding sequence in the *NWC* promoter is shared with other transcription factors (e.g., the TGGGAAT of Ikaros [21]), we sought to confirm the involvement of ZFP143 in the activation of the *NWC* promoter by performing competition experiments with a dominant-negative form of ZFP143. We co-transfected reporter vectors containing the −119/+125 and +28/−258 fragments of the promoter (for activity testing in the *NWC* and *RAG-2* directions, respectively) with a vector expressing the DNA-binding domain (DBD) of ZFP143. If ZFP143 binds to the promoter, then overexpressed ZFP143-DBD should outcompete the endogenous protein for the binding site and decrease the expression of the reporter gene. As shown in Figure 3D, overexpression of ZFP143-DBD reduced the *NWC* promoter activity in both directions, while overexpression of mutant ZFP143-DBD had no significant effect. Also, overexpression of ZFP143-DBD did not affect the activity of a promoter that lacked the ZFP143 binding site (the SV40 promoter in pGL3-Prom; used as a control). Together, these results indicate that ZFP143 binding is essential for activating the *NWC* promoter in both directions.

### The *NWC* Promoter Activates the Transcription of RAG-2 in Non-lymphoid Cells

**Figure 4:**
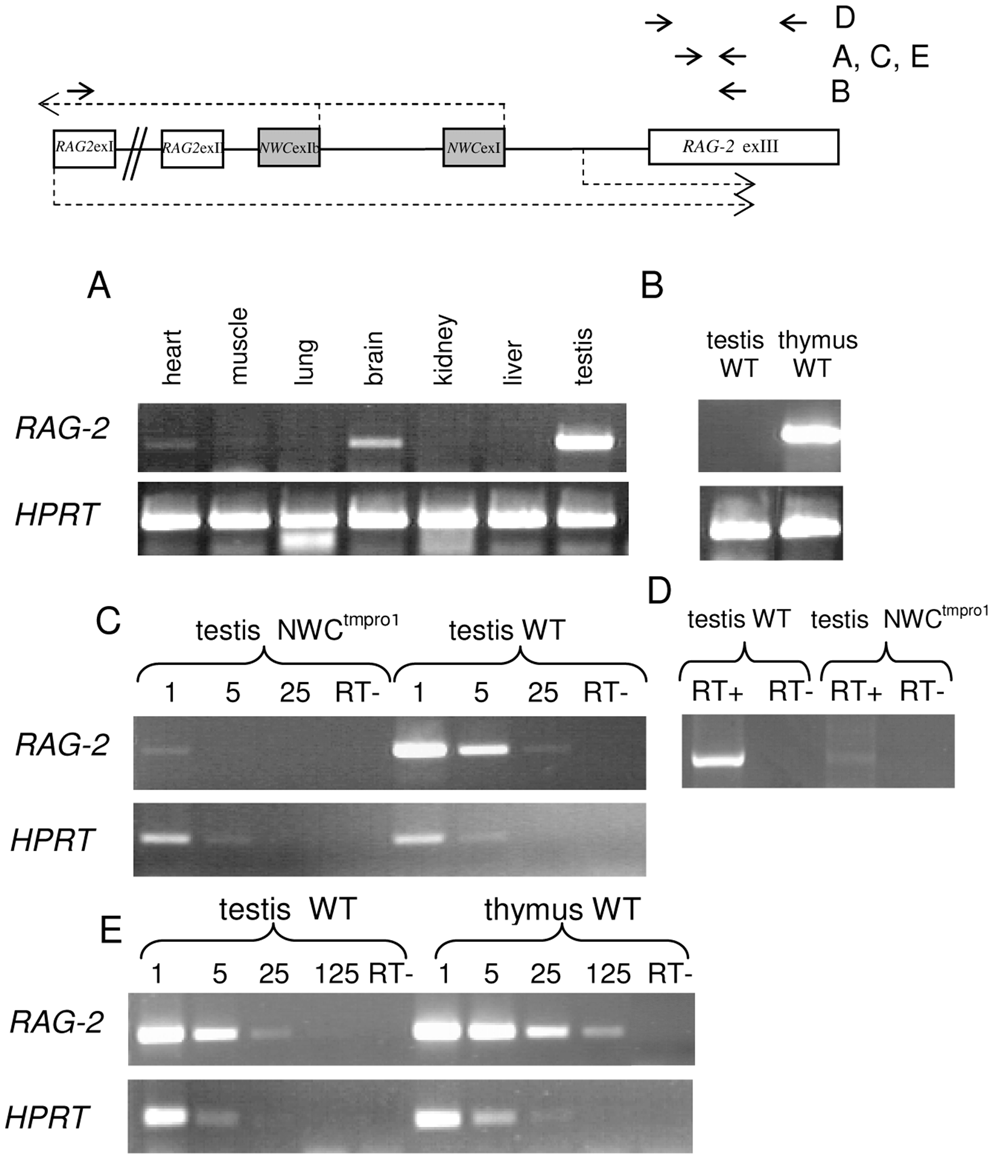
RT-PCR analysis of *RAG-2* **expression.** RT-PCR analysis of *RAG-2* expression in (A) murine tissues; (C) and (D) testis of wild-type and NWC^tmpro1^ mice; (B) and (E) thymus and testis of wild-type mice. The positions of the primers (solid horizontal arrows) used for each analysis are indicated. Primers were designed to distinguish *RAG-2* lymphoid-specific transcripts (B) from total *RAG-2* expression including non-lymphoid transcripts (A, C, E) and the full-length *RAG-2* open reading frame (D). Numbers indicate the utilized cDNA -fold dilution. Amplification of the *HPRT* gene was used for normalization of the cDNA quantity. The same cDNA was used in (C) and (D) and corresponding *HPRT* amplification is shown in panel (C).

After demonstrating that the *NWC* promoter has bidirectional activity, we searched for evidence of *RAG-2* transcription in non-lymphoid cells. Using cDNA prepared from various murine tissues, we performed RT-PCR with primers that amplified a fragment of the third exon of *RAG-2*. The experiment was carefully controlled for the presence of contaminating genomic DNA, as the utilized primers were not intron-spanning. Under standard PCR conditions (30 cycles) an amplification product was observed from testis. When we increased the number of PCR cycles to 35, we detected weak reaction products in the brain and some other tissues (Fig. 4A). The specificity of the PCR reaction was confirmed by sequencing of the amplification product.

To exclude the possibility that this expression of *RAG-2* was due to leakiness of the *RAG-2* promoter, we performed RT-PCR on cDNA from testis with primers complementary to the first and third exons of *RAG-2*, and thus capable of detecting only the standard lymphoid version of the *RAG-2* transcript. We did not detect any reaction product (Fig. 4B). Further evidence pointing to the involvement of the *NWC* promoter in activating the transcription of *RAG-2* was provided by a knockout mouse harboring a deletion of the promoter and first exon of the *NWC* gene (Fig. 1) [17]. Expression of the third exon of *RAG-2* was 25 times lower in the testis of knockout mice compared to the wild-type control, as determined by semiquantitative (Fig. 4C) and real-time (not shown) RT-PCR analysis. This is most likely due to residual (apparently also bidirectional) activity of the secondary *NWC* promoter located downstream of the knockout region. A comparable analysis using primers encompassing the entire open reading frame of *RAG-2* yielded similar results (Fig. 4D). To estimate the expression level of non-lymphoid *RAG-2* expression in testis, we compared it with the level of *RAG-2* expression in the thymus. Semiquantitative (Fig. 4E) and real-time RT-PCR analysis using primers common to both (thymus and testis) forms of *RAG-2* transcripts revealed that the expression of *RAG-2* in the testis was 5–10 times lower than that in the thymus.

The expression profile of the non-lymphoid *RAG-2* transcript is consistent with the known expression profile of *NWC,* which is about 10-fold higher in testis than in other tissues [15], [17]. When we compared the expression levels of *RAG-2* and *NWC* transcripts in the testis using real-time RT-PCR, we found that expression of *NWC* was 9-fold higher than that of *RAG-2*. In contrast to the results of our DLR analysis, these findings indicate that the activity of the endogenous *NWC* promoter may not be similar in both directions.

## Discussion

The main results of the present study are the demonstrations (*in vitro* and *in vivo*) that the *NWC* promoter functions as a bidirectional promoter, binds the ZFP143 transcription factor, and activates transcription of the *RAG-2* protein-encoding exon in non-lymphoid cells. We did not exclude the possibility that Ikaros (another transcription factor specific for similar binding sites) may also regulate the *NWC* promoter. However, unlike the ubiquitously expressed ZFP143 [22], Ikaros is a lymphocyte-specific factor and thus cannot regulate *NWC* expression in non-lymphoid cells. The *NWC* promoter is silenced in lymphocytes, prompting us to speculate that Ikaros could be responsible for the lymphocyte-specific inactivation of *NWC* by competing with ZFP143. Future work will be needed to test this hypothesis.

The expression of the non-lymphoid *RAG-2* transcript was significant in the testis, where it was only 5 to 10 times lower than that in developing thymocytes. The expression level of *RAG-2* correlated with that of *NWC*, which is also higher in testis than in other tissues [17]. However, the expression of the non-lymphoid *RAG-2* transcript is lower than that of *NWC*, potentially suggesting that the *NWC* promoter is not equally active in both directions**.** Another explanation for this observation could be that the primary *RAG-2* transcript is devoid of introns and therefore perhaps less stable than the *NWC* transcript. It has been shown that the presence of introns can enhance the expression of primary transcripts [23] and increase the efficiency of translation, because splicing near the 5′UTR promotes export of RNA from the nucleus to the cytoplasm [24]. Thus, the lack of introns in the *RAG-2* transcript might contribute to our inability to detect RAG-2 protein by Western blotting of testis tissues (not shown). Previously, the possibility of *RAG-2* expression in the central nervous system was put forth in a study showing expression of *RAG-1* transcripts in neurons, but the evidence was not convincing [14]. The authors speculated on the possible role of RAG-1/RAG-2 recombinase or an active *RAG-1* gene product in the nervous system, but to our knowledge, none of these possibilities received experimental support. Thus, our demonstration of *RAG-2* transcript expression in non-lymphoid tissues, including brain, may raise new speculations on the presence of a functional recombinase in the nervous system. However, we would consider such a discussion to be premature. For us, the current questions are: whether the bidirectional activity of the *NWC* promoter and *RAG-2* transcription itself plays any physiologically important role in non-lymphoid cells; whether it is simply a meaningless consequence of a not-so-strict control of transcription within the *RAG/NWC* locus; or, more interestingly, whether it represents the remnants of an ancient event involved in the evolutionary “domestication” of the *RAG*s. The popular hypothesis on the origin of *RAGs* proposes that the transposon(s) containing the *RAG* transposase infected a germ cell of an ancestor of the jawed vertebrates (or deuterostomes), ultimately allowing for the development of lymphocytes [25]–[27] and we suggested that *NWC* could have played an important role in the evolution of adaptive immunity by controlling *RAG* expression. We previously proposed that the transcription of *NWC* activated by its own promoter, which runs through the whole *RAG* locus control region, could negatively regulate *RAG* expression in non-lymphocytes [28] and reduce the potential danger that the RAG recombinase can pose to genome integrity [29].

One of the other open questions regarding the initial integration and “survival” of the *RAG* transposon is how it was able to avoid host-mediated epigenetic silencing, which is the fate of most transposons upon entering the genome. The bidirectional activity of the *NWC* promoter and the other features documented in this study suggest a possible answer. It was recently shown that, as in the case of *RAG* and *NWC*, many transposon-derived genes that are still functional in their host genomes are localized in the immediate vicinity of the 5′ end of host genes that are controlled by CpG island-containing bidirectional promoters [30]. The open chromatin structure of such an arrangement would favor the integration and survival of transposon-derived genes; their expression would be ensured via the activity of the host gene promoter; and, most importantly, any attempt at silencing the transposon gene through methylation of the CpG island of the bidirectional promoter would also affect the expression of the host gene. Given that many CpG island-containing promoters control housekeeping genes, such inactivation could be fatal for the host organism. The strong evolutionary conservation and ubiquitous across-species and -tissue expression of *NWC* [15], [31] indicates that it is likely to represent an important housekeeping gene, and we would like to suggest that the integration and survival of the RAG transposon was facilitated by the bidirectional activity of the *NWC* promoter. This assumption is further supported by the strong evolutionary conservation of the *NWC* promoter structure, as well as our finding that the presence and localization of the ZFP143 binding elements, which are crucial for the bidirectional activity of the *NWC* promoter in mice, are similar among the mammalian (human, murine and dog), amphibian (*Xenopus tropicalis*) and fish (*Latimeria chalumnae*) *NWC* promoters. This strongly suggests that the bidirectionality of the *NWC* promoter is an evolutionarily conserved feature, at least in the case of the jawed vertebrates. Notably, orthologs of *NWC* can also be found in some invertebrate species, including the sea urchin *Strongylocentrotus purpuratus,* which is the only invertebrate in which both *RAG* genes have been found [25]. However, the *NWC* and *RAG* loci are not linked in *S. purpuratus*. This difference in the localization of the *RAGs* in the sea urchin genome versus those of the jawed vertebrates may suggest that two independent integration events occurred, or that a secondary transposition event led to the separation (in echinoderms) or joining (in a common ancestor of the jawed vertebrates) of the *RAG* and *NWC* loci. Further studies, including investigations into the mechanisms responsible for regulating *NWC* expression in invertebrates, will be needed to clarify this issue.

To summarize, the present study shows that the *NWC* promoter has bidirectional activity that results in the transcription of *RAG-2* in nonlymphoid cells. This novel observation suggests a possible mechanism that may have protected the RAG transposase from inactivation during its integration into the genome of a vertebrate ancestor.

## Materials and Methods

### RNA Extraction and Semiquantitative Real-time RT-PCR

All procedures using animals were reviewed and approved by the First Local Ethical Commission for Animal Experimentation, at the Institute of Immunology and Experimental Therapy (Wroclaw, Poland; permit number 13/2009).

RNA was isolated from C57/BL6 mice, NWC knockout mice (NWC^tmpro1^ [17]), or cultured cell lines using the TRIzol Reagent (Invitrogen). Three micrograms of total RNA was digested with RNase-free DNase I (Promega, Fermentas) and reversed transcribed with SuperScript III Reverse Transcriptase (Invitrogen) and random hexamer oligonucleotides at 50°C. PCR reactions was performed under the following conditions: denaturation at 95°C for 3 min, followed by 30 cycles of denaturation at 94°C for 30 s, primer annealing at 52°C for 30 s, and extension at 72°C for 1 min, followed by a final extension at 72°C for 3 min. Equal amounts of total RNA were amplified without reverse transcription to test for contamination with genomic DNA (RT- control).

Real-time RT-PCR was performed on a DNA Engine Opticon 2 apparatus (BioRad) using the Maxima SYBR Green qPCR Master Mix (Fermentas). The thermal-cycling conditions comprised an initial denaturation step at 95°C for 10 min, followed by 40 cycles of 15 s of denaturation at 95°C, 30 s of annealing at 55°C, and 30 s of elongation at 72°C. Expression values were normalized with respect to that of *HPRT*. Standard curves for each primer pair were prepared by serial 5-fold dilutions of the template cDNA followed by determination of reactions efficiencies.

The following primer pairs were used:

*HPRT*: HprtF GCTGGTGAAAAGGACCTCT (forward).

HprtR CACAGGACTAGAACACCTGC (reverse).

*RAG-2 lymphoid transcript*: RAG2exIF TTCAGAGAGGGATAAGCAGC (forward).

RAG2 630- GTAGAAGGCATGTATGAACGTC (reverse).

*RAG-2 non-lymphoid transcript*: RAG-2exIIIF TCTCAAACTGAAGCCTGCAA (forward).

RAG2exIIIR GCACACCCAAATTCAAAATCT (reverse).

*RAG-2 ORF*: RAG2cdsF ATGTCCCTGCAGATGGTAAC (forward).

RAG2cdsR TTAATCAAACAGTCTTCTAAGGAAGGA (reverse).

*NWC:* NWCexIF GGCTGTTTGACTGGATAAGG (forward).

NWCexIVR CACCTTGAAAGGAACTCCCA (reverse).

### Plasmid Construction

*NWC* promoter fragments were PCR amplified from genomic bacterial artificial chromosomes (BACs) containing the *RAG/NWC* locus (RP23-111E15). All utilized oligonucleotides contained *BglII* 5′ overhangs. The amplified fragments were digested with *BglII* and cloned into the *BglII* site of the pGL3-Basic vector (Promega). The orientation of the cloned fragments was verified by PCR. Constructs containing internal deletions of the *NWC* promoter (M1–M6) and point mutations of the ZFP143 binding sites were generated by PCR ligation [16] and cloned into the *BglII* site of pGL3-Basic. To construct the ZFP143 DNA-binding domain expression vector, a fragment encoding the DNA-binding domain of ZFP143 (amino-acid positions 235–488) was amplified from murine testis cDNA with oligonucleotides containing *EcoRI* and *XhoI* overhangs, and cloned into the pCDNA3.1 vector. The mutant form of the ZFP143 DNA-binding domain was generated using PCR ligation and substituting the cysteine-encoding codons with alanine-encoding codons in the third and seventh zinc finger domains of the ZFP143 DNA-binding domain. To obtain a vector expressing His-tagged full-length ZFP143 (pLVX-ZFP143His), a polyhistidine tag-encoding sequence was added to the ZFP143 cDNA during the PCR reaction and the construct was cloned into *EcoRI* sites of the pLVX-IRES-ZsGreen1 vector. Finally, to obtain the constructs for bacterial expression of recombinant N-terminal His-tagged proteins, the cDNAs encoding the ZFP143 DNA binding domain or its mutant form were cloned into the *EcoRI/XhoI* sites of the pET28a vector. All constructs were verified by sequencing, and all primer sequences are available upon request.

### Recombinant Protein Purification

Vectors encoding N-terminal His-Tag ZFP143-DBD or ZFP143DBDmut proteins were transformed into *E. coli* strain BL21. Overnight cultures were diluted 500–fold, grown at 37^O^C until the OD_600_ reached 0.5, and then incubated for 1.5 h at 4^O^C. The bacteria were induced with 0.25 M IPTG, ZnCl_2_ was added to a final concentration of 100 uM, and the incubation was continued for 5 h at room temperature. After centrifugation, the bacterial pellets were resuspended in buffer A (10 mM imidazole, 50 mM Tris-HCl, pH 8.0, 300 mM NaCl, 10% glycerol and 0.05% Tween 20). After centrifugation, the supernatant was applied to HisPur Ni-NTA resin and incubated for 1 h at 4^O^C. The resin was washed several times with buffer B (20 mM imidazole, 50 mM Tris-HCl, pH 8.0, 300 mM NaCl, 10% glycerol and 0.05% Tween 20). The recombinant proteins were eluted with buffer C (250 mM imidazole, 50 mM Tris-HCl, pH 8.0, 300 mM NaCl, 10% glycerol and 0.05% Tween 20), and dialyzed against a buffer containing 20 mM HEPES, pH 7.9, 0.2 mM EDTA, 20% glycerol, 100 mM KCl, 1 mM DTT, 10 uM ZnCl_2_ and 1 mM PMSF. Buffers A, B and C were additionally supplemented with 20 uM ZnCl_2_, 30 uM MgCl_2_, 50 uM CaCl_2_ and 1 mM PMSF.

### Electromobility Shift Assay (EMSA)

The probe containing the *NWC* promoter was obtained by amplifying the DNA fragment encompassing nucleotides −119/+12 relative to the *NWC* transcriptional start site using a digoxigenin-labeled primer. Cells were transfected with pLVX-ZFP143His. After incubation for 48 hours, the cells were washed with ice-cold buffer (10 mM HEPES, pH 7.9, 1.5 mM MgCl_2_, 10 mM KCl and 0.5 mM PMSF) and homogenized, and nuclei were pelleted at 1000xg at 4°C. Proteins were extracted in buffer containing 20 mM HEPES, pH 7.9, 1.5 mM MgCl_2_, 420 mM KCl, 0.2 mM EDTA, 0.5 mM DTT, 25% glycerol and 0.5 mM PMSF. The binding reaction was performed for 20 minutes on ice in 20 ul of reaction mix containing binding buffer (for recombinant proteins, 20 mM HEPES, pH 7.9, 0.2 mM EDTA, 20% glycerol, 100 mM KCl, 1 mM DTT and 10 uM ZnCl_2_; for nuclear extracts, 10 mM HEPES, pH 7.5, 5 mM MgCl_2_, 10 mM KCl, 20 uM ZnCl_2_, 5% glycerol and 0.1% Nonidet P40), 150 ng of purified recombinant protein or 10 ug of nuclear extract, 1 ug poly(dI-dC) and 0.035 pmol of probe. Where noted, a 100-fold molar excess of unlabeled competitor oligonucleotide or anti-His antibody was added. The samples were subjected to 6% polyacrylamide gel electrophoresis (PAGE) at 4°C, the resolved proteins were transferred to a nylon membrane, and the results were detected with anti-digoxigenin antibodies [Roche], according to the manufacturer’s recommendations.

### Cell Culture, Transfection and Dual Luciferase Reporter Assay

NIH3T3 and HEK293T cells were cultured in DMEM (Sigma-Aldrich) supplemented with 10% FBS (Invitrogen). A total of 2 × 10^5^ NIH3T3 or HEK293T cells were transfected with 500 ng of firefly luciferase-encoding reporter plasmid and 50 ng of renilla luciferase-encoding plasmid (pRL-TK; Promega) using the MetafectenePro reagent (Biontex). In experiments using ZFP143-encoding expression vectors, cells were transfected with 500 ng of the ZFP143 expression vector, 100 ng of firefly luciferase-encoding reporter plasmid and 50 ng of pRL-TK. A dual-luciferase reporter (DLR) assay was performed 24 h after transfection using the Dual-Luciferase Reporter Assay System (Promega). Cells were lysed with 100 µl of passive lysis buffer, and 15 µl of cell lysate was used for each assay. The data are presented as the ratio of firefly luciferase activity (FLU) to renilla luciferase activity (RLU). One-way ANOVA followed by the Tukey-Kramer post-hoc test were used for statistical analysis.

### RNA Ligase-mediated 5′-RACE

For determination of the transcription initiation site, RNA ligase-mediated 5′ rapid amplification of cDNA ends (5′-RACE) was performed using the FirstChoice RLM-RACE kit (Ambion). After ligation of the 5′ RACE adapter, RNA was reverse-transcribed with a firefly luciferase gene-specific primer (TCCAGCGGATAGAATGGCGC). Nested PCR was performed using luciferase gene-specific primers (GCTCTCCAGCGGTTCCATCT and GGCGTATCTCTTCATAGCCTTATGC) in conjunction with primers complementary to the 5′ RACE adapter. The resulting PCR products were cloned into the pGEM-T easy vector (Promega) and subjected to sequencing.
